# Supplementary material for: PD-L1+ and XCR1+ dendritic cells are region-specific regulators of gut homeostasis
Source: Nat Commun. 2021 Aug 13;12:4907. doi: 10.1038/s41467-021-25115-3 (PMC8363668; doi:10.1038/s41467-021-25115-3)
Supplement: Supplementary file 1 — Supplementary Information [file 41467_2021_25115_MOESM1_ESM.pdf]

Supplementary Figure 1

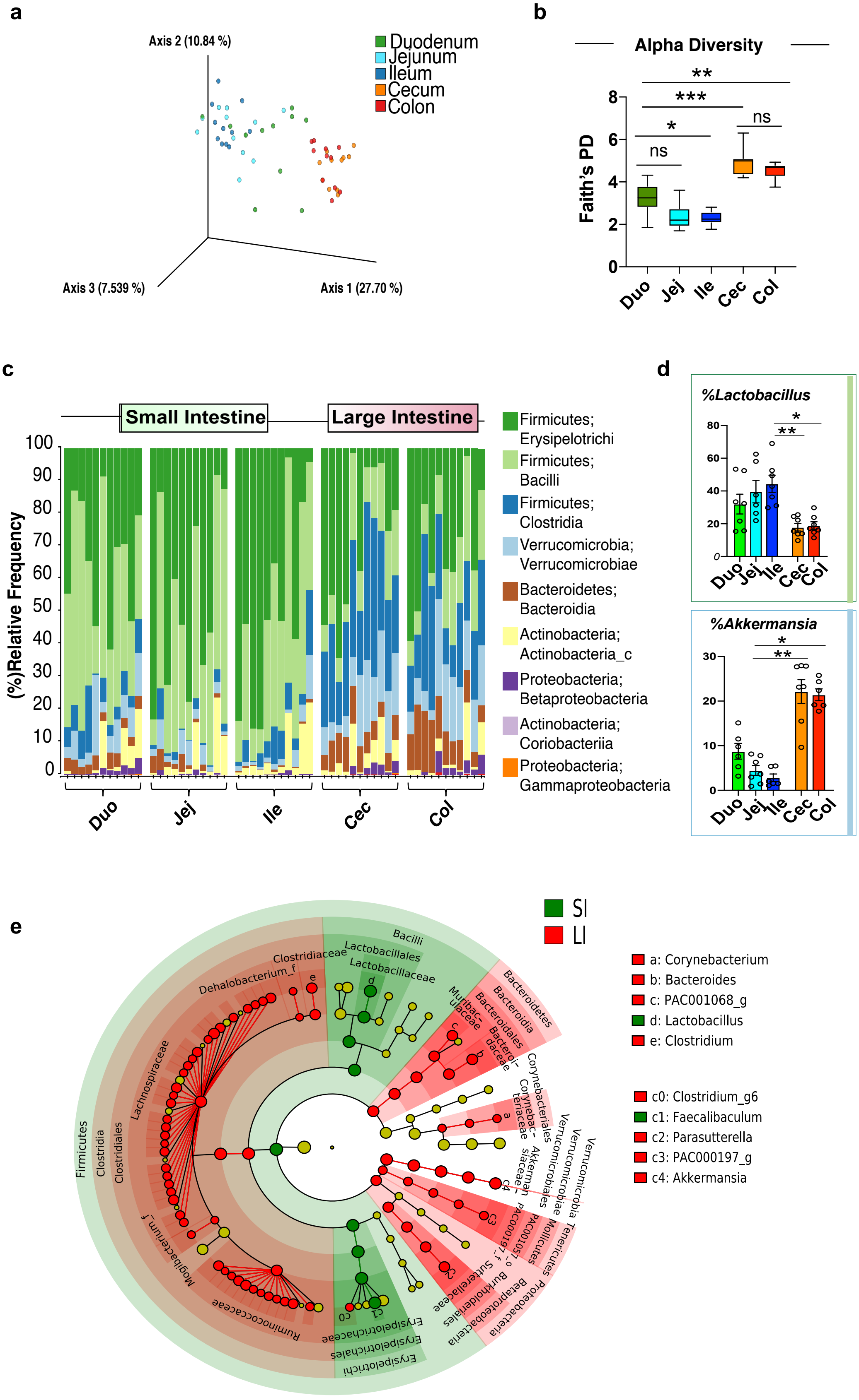

Supplementary Figure 1. Regional distribution of microbiota. Fecal microbiota samples were collected from duodenum (Duo), jejunum (Jej), ileum (Ile), cecum (Cec) and colon (col) of 8-week-old naïve C56Bl/6J mice N=7. (a)16S rRNA-sequenced microbiome clustered using principal coordinate Analysis (PCoA) from Unweighed UniFrac distances. (b) Alpha diversity boxplots representing Faith's Phylogenetic Diversity comparison between regions of the gut. One-way ANOVA followed by Tukey post-hoc test for multiple comparisons (c) Relative abundance of bacterial taxa in different regions of the gut at the class level. (d) Prevalence of species Lactobacillus and Akkermansia in different regions of the gut. (e) Cladogram representing all taxa detected at >0.1%, shown at the Kingdom phylogenetic level through the genus level LEfSe $p < 0.05$. A yellow circle depicts taxa present, but not enriched. Red circles are enriched in large intestine and green circles represents small intestine. The size of the circle corresponds to the population of each taxa. Boxes and bars represent mean+SEM . Unless specified, Kruskal-Wallis one-way analysis followed by Dunn's test for multiple comparisons was used for microbiome analysis. Small Intestine (SI); Large Intestine (LI).

Supplementary Figure 2

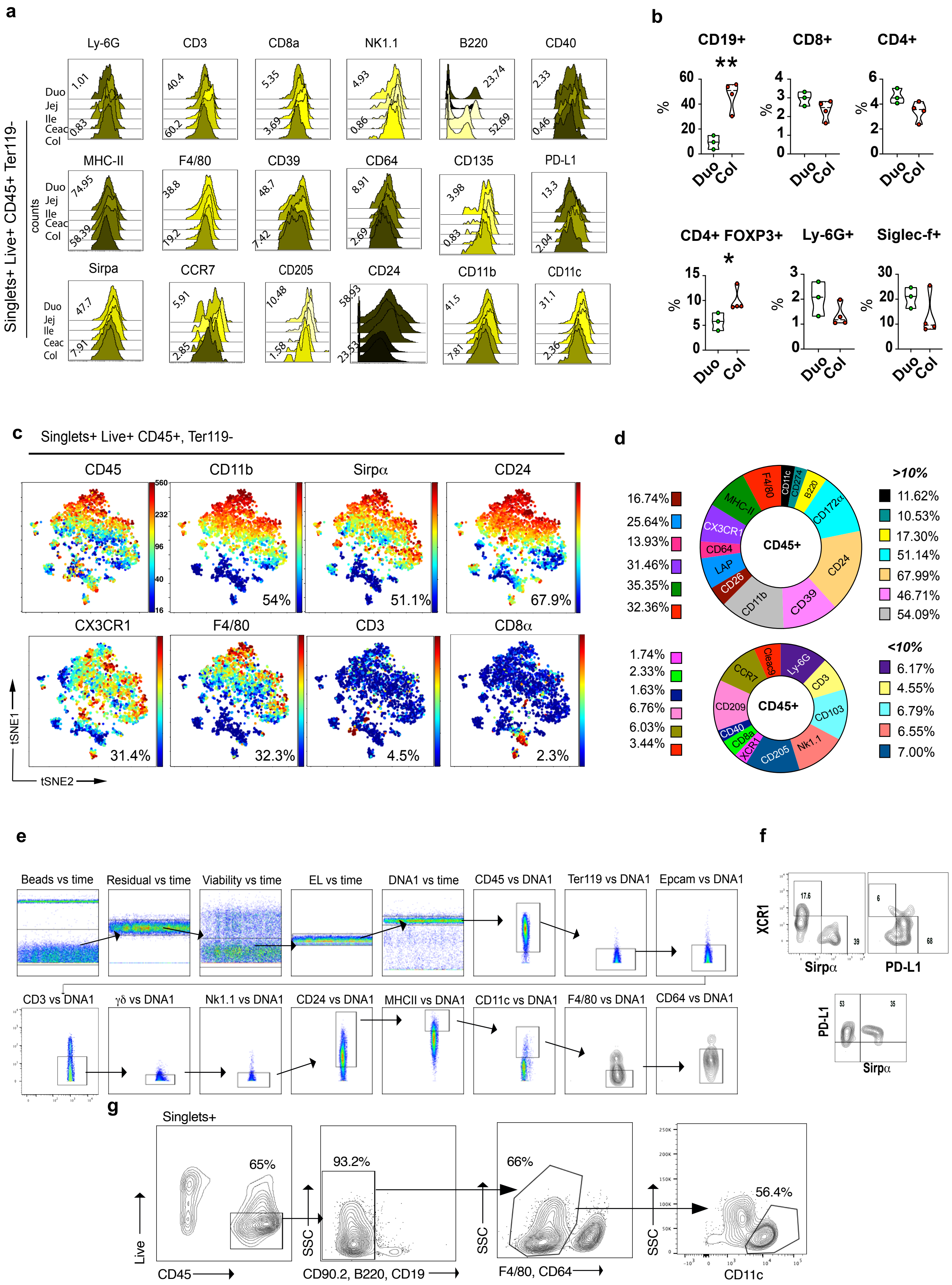

Supplementary Figure 2. Immune cells are differentially distributed in gut compartments. (a) Mass cytometry (CyTOF) representative histograms from different regions of the gut using concatenated data. Samples were gated on singlets, live, $CD45^+$ and $Ter119^-$ cells. (b) Flow cytometry analysis of $CD19^+$, $CD8^+$, $CD4^+$, $Foxp3^+$, Ly-$6G^+$, Siglec-f^+ cells gated on $singlets^+$, $live^+$, $CD45^+$ cells from naïve WT mouse LP. N= at least 3 over at least 3 independent experiments. Unpaired two-tailed Student's t test. * $p<0.05$, **$p<0.01$. (c) Mass cytometry t-SNE plots of stomach cells gated on singlets, live, $CD45^+$ and $Ter119^-$ cells. (d) Pie chart showing percentage of several markers on $CD45^+$ gated cells isolated from stomach. N=10 mice/region of the gut. Bars represent mean+SEM (e-f) gating strategy for DC CyTOF analyses in figure 1a-c and 2f, (g) gating strategy for flow cytometry experiments including immunophenotyping experiments and DC sorting for RNA-seq, RT-PCR and coculturing experiments in figures 2a-e, 2f, 3a-b, 4-a-f. Duodenum (Duo), Jejunum (Jej), Ileum (Ile), Cecum (Cec) and Colon (col).

Supplementary Figure 3

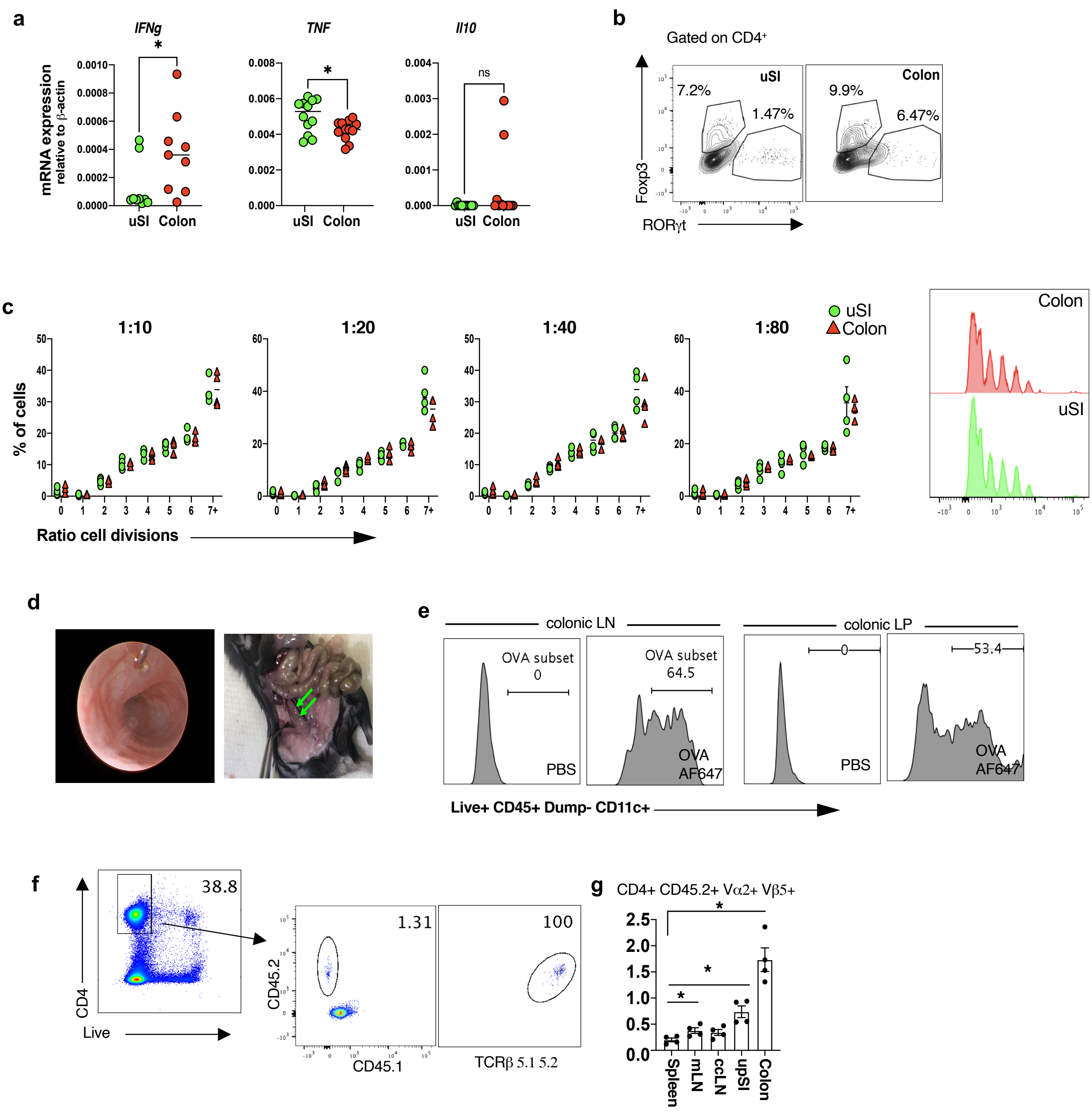

Supplementary Figure 3. T cell differentiation in vitro and in vivo by intestinal DC. (a) OT-II naïve T cell cocultured with upper Small Intestine (uSI) or colonic DCs for 7 days in the presence of OVA peptide[323-339] were sorted and gene expression of *Ifng*, *Tnf* and *Il10* was measured by RT-PCR. (b) Flow cytometry dot plots showing Foxp3 and RORγt in T cells cocultured with uSI DC for 7 days. (c) OT-II$^+$ T cell proliferation with different DC:T cell ratios in coculture of uSI or colonic DCs for 7 days. (d) Representative picture of intra-colonic OVA injection and colonic lymph nodes. (e) OVA$^+$ DCs in colonic lymph node and colonic LP 4h after OVA injection showing that DCs efficiently uptake intracolonic OVA. Cells were gated on singlets$^+$, live$^+$, Dump$^-$ (CD3, B220, CD19) F4/80$^-$CD64$^-$CD11c$^+$. (f) FACS dot plots showing gating strategy for adoptive transferred T cells from colonic LP. (e) Distribution of Vβ2$^+$Vβ5$^+$ OT-II T cells in different organs 7 days after continuous feeding of OVA (8g/L) and intra-colonic OVA (2.4 mg) injection. N=4. Dots and bars represent mean+SEM. One-way ANOVA, * p<0.05. Lymph node (LN); mesenteric LN (mLN); (iliac+caudal+cecum) (ceLN); Colon (Col).

Supplementary Figure 4

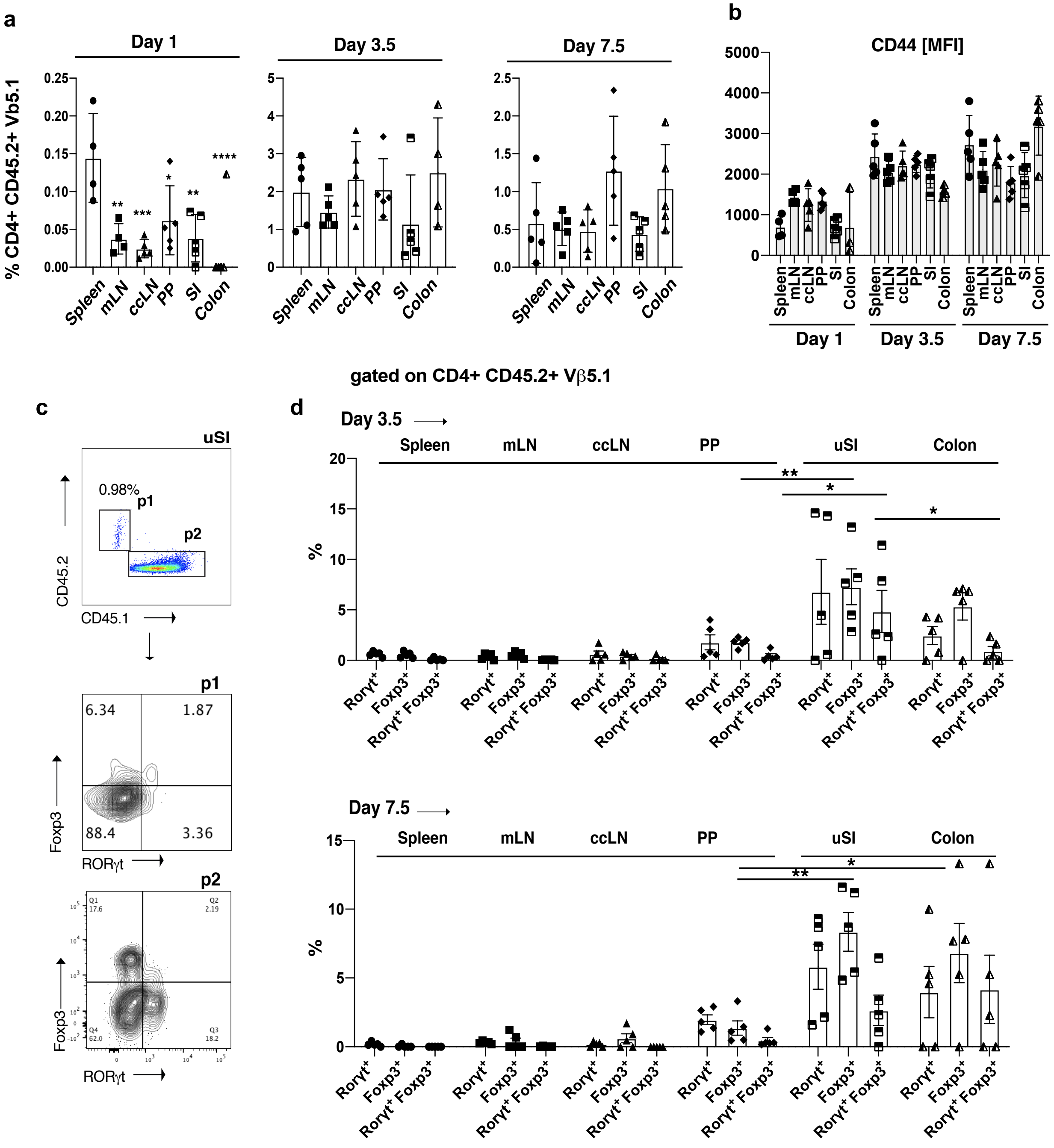

Supplementary Figure 4. Intestinal DCs drive T cell differentiation in situ. Recipient mice (CD45.1) received 2.5 x 10^6 naïve T cells from OT-II mice (CD45.2) followed by intrarectal injection of OVA (16h hours after T cell transfer) and continuous feeding of OVA (8g/L) throughout the experiment. Mice were sacrificed after 24h (1 day), 84h (3.5 days) and 180h (7.5 days) post cell transfer. Cells were harvested from spleen, Peyer's patches (PP), lower gut draining lymph nodes (iliac+caudal+cecum) (ccLN), mesenteric lymph node (mLN), upper small intestine (uSI) and colon. (a) Percentage of $CD4^+$ $CD45.2^+$ $V\beta5.1^+$ in each site and time point. (b) Median of Fluorescence Intensity (MFI) of CD44 gated on $CD4^+$ $CD45.2^+$ $V\beta5.1^+$ cells in each site and time point. (c) Representative flow cytometry dot plot showing Foxp3 and RORγt staining in $CD4^+$ transferred (CD45.2) and endogenous (CD45.1) cells in the uSI 7.5 days after adoptive transfer. (d) Percentage of Foxp3 and RORγt single and double positive population in $CD4^+$ $CD45.2^+V\beta5.1^+$ cells in each site and time point. N=5 mice per time point. Experiment was performed in duplicate. Bars represent mean+SEM. One-Way ANOVA followed by Tukey post-hoc test for multiple comparisons on each T cell population. * $p<0.05$. ** $p<0.01$ are only displayed for relevant analysis. .

Supplementary Figure 5

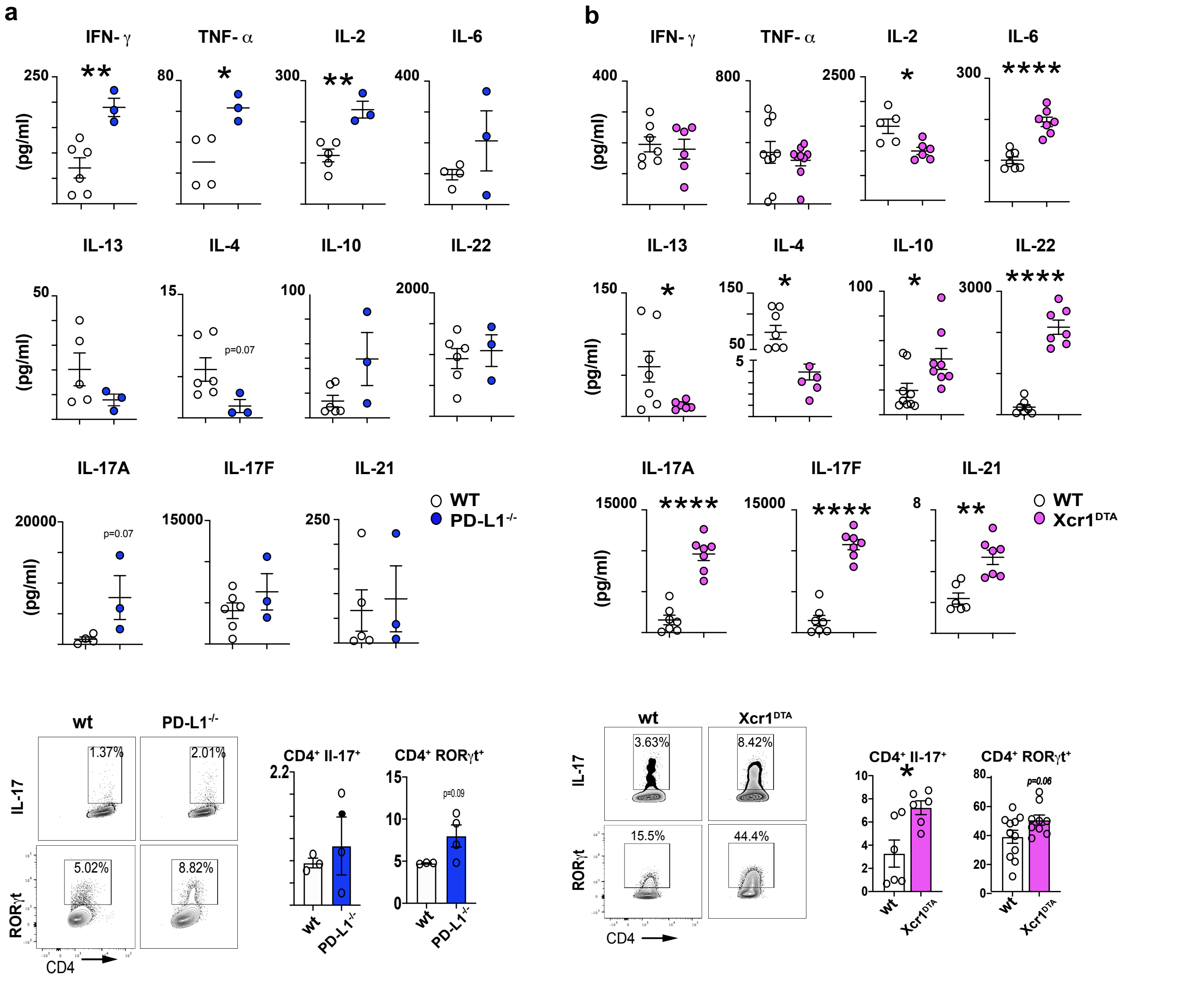

Supplementary Figure 5. T cells differentiation with DCs from *Pd-l1-/-* and XCR1^{DTA} mice. (a) Cytokine quantification by Legendplex from supernatants of DC sorted from upper Small Intestine (uSI) (duodenum and proximal jejunum) from *Pd-l1-/-* mice cultured with naïve OT-II^{+} CD4^{+} T cells for 7 days in the presence of OVA peptide$^{323-339}$ and intracellular staining of cultured T cells after differentiation (day 7) showing IL-17A and RORγt expression. (b) Cytokine quantification by Legendplex from supernatants of DC sorted from colon of XCR1^{DTA} mice cultured with naïve OT-II^{+} CD4^{+} T cells for 7 days in the presence of OVA peptide$^{323-339}$ and intracellular staining of cultured T cells after differentiation (day 7) showing IL-17A and RORγt expression. N= at least 3 over at least 2 independent experiments. Dots and bars represent mean+SEM. Two-tailed unpaired Student's t test * p<0.05, ** p<0.01, ****p<0.0001. Wild-type (WT), **p<0.01 are only displayed for relevant analysis.

Supplementary Figure 6

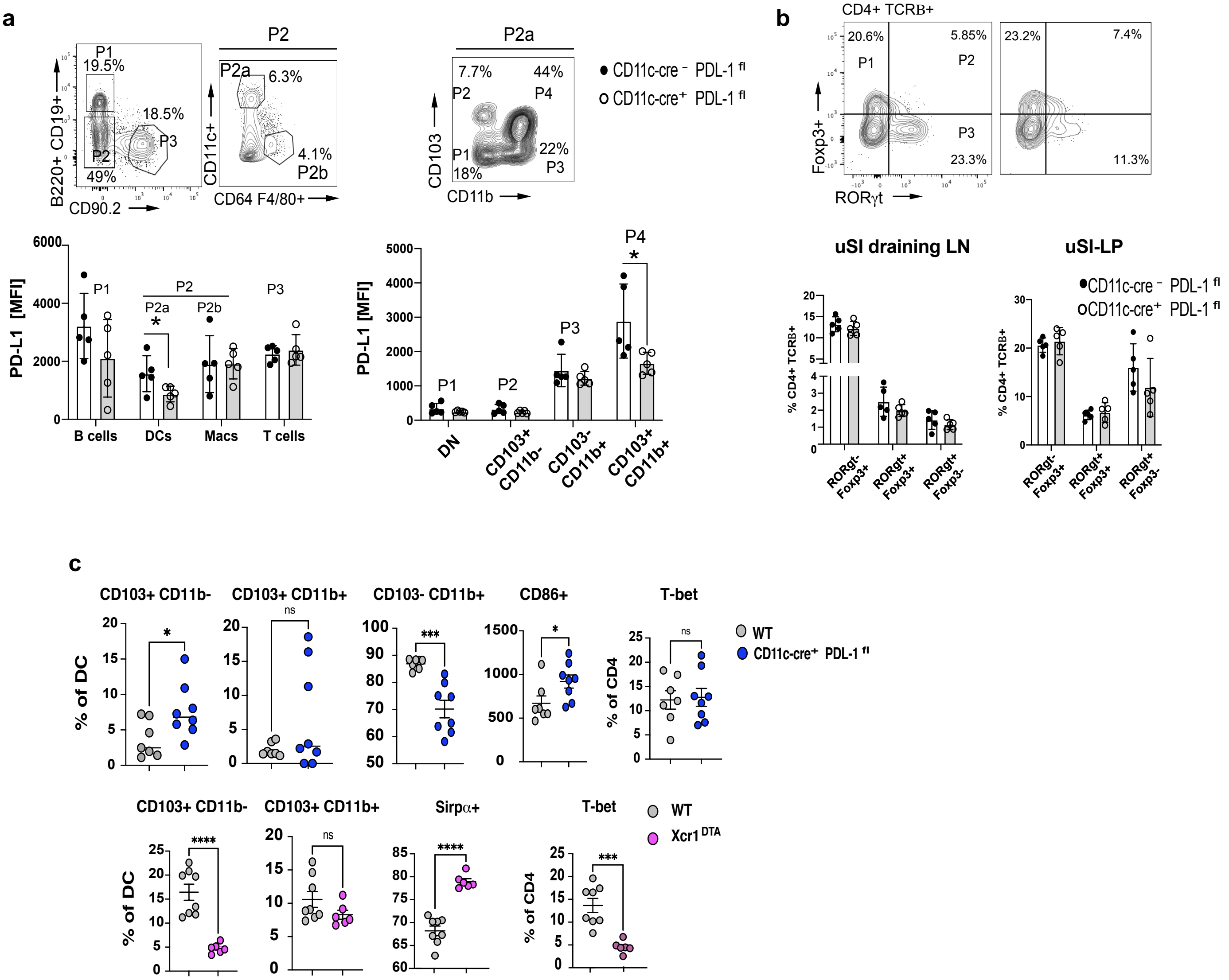

Supplementary Figure 6. Characterization of upper Small Intestine LP from CD11c^{Cre} Pd-l1$^{flox/flox}$ mice. (a) Representative dot plots and gating strategy of LP B, T cells, macrophages and DCs (CD103$^+$ CD11b$^+$ and CD103$^+$ CD11b$^-$) showing PD-L1 Median Fluorescence Intensity (MFI) in each LP cell type in steady-state conditions. (b) Foxp3 and RORγt expression in TCRβ$^+$ CD4$^+$ T cells in upper Small Intestine (uSI) LP and uSI draining lymph nodes in CD11c^{Cre}Pd-l1$^{flox/flox}$ mice mice versus littermate controls in steady-state condition. N=5. Bars represent mean+SEM. Unpaired two-tailed Student's t test * $p<0.05$. (c) Flow cytometry quantification of CD86, CD103, CD11b and Sirpα on DCs and T-bet in CD4$^+$ T cells isolated from uSI of CD11c^{Cre}Pd-l1-$^{flox/flox}$ mice and littermate controls after 5-FU-induced mucositis. N=6-8. Dots represent mean+SEM. Unpaired two-tailed Student's t test * $p<0.05$, ** $p<0.01$*** $p<0.001$ ****$p<0.0001$.

Supplementary Figure 7

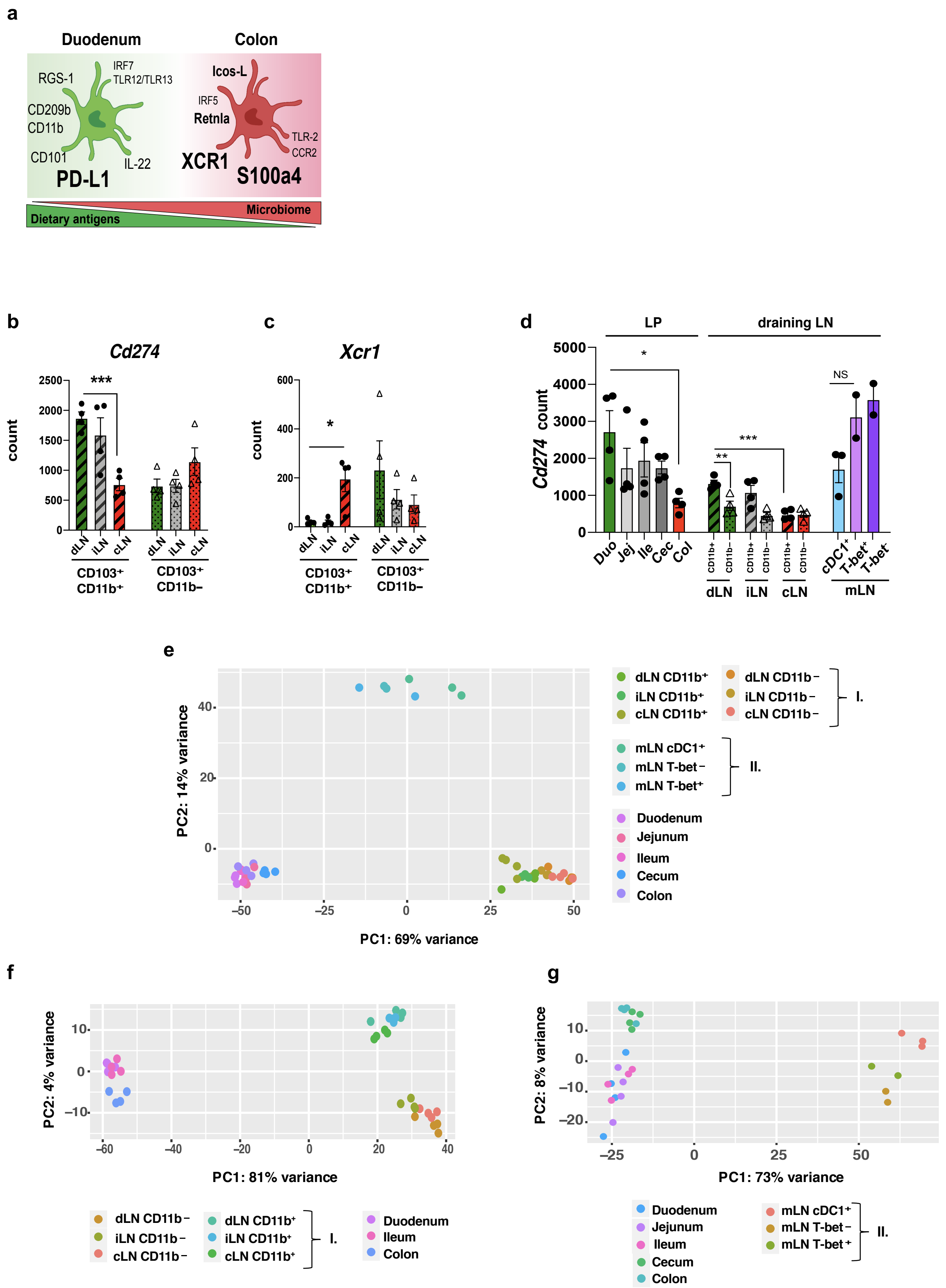

Supplementary Figure 7. RNA-seq comparison between intestinal DC and lymph node DC database. (a) Summary of our findings of DC transcriptome showing upregulation and downregulation of genes found in RNA-seq analysis from DCs sorted from duodenal and colonic LP of 10 8-week old naïve C57BL/6J mice. (b-g) RNA-seq data from draining lymph node DC database from (I.) (Esterhazy et al., 2019) (GSE121811) and (II.) (Brown et al., 2019) (GSE130201) were compared to LP RNA-seq data from DCs sorted from duodenum, jejunum, ileum, cecum and colon. (b) *Cd274* and (c) *Xcr1* counts in duodenal (dLN), ileal (iLN) and cecal-colonic (cLN) draining lymph nodes from $CD103^{+}CD11b^{+}$ and $CD103^{+}CD11b^{-}$cells (GSE121811). (d) *Cd274* counts in LP DCs and draining LN database (I.) and (II.). N= at least 4. Bars represent mean+SEM. One-Way ANOVA followed by Tukey post-hoc test for multiple comparisons. * $p<0.05$, **$p<0.01$, ***$p<0.001$. (e-g) Principal component analysis (PCA) of RNA-seq comparisons. Source data are provided as a Source Data file.
