# Supplementary material for: PD-L1+ and XCR1+ dendritic cells are region-specific regulators of gut homeostasis
Source: Nat Commun. 2021 Aug 13;12:4907. doi: 10.1038/s41467-021-25115-3 (PMC8363668; doi:10.1038/s41467-021-25115-3)
Supplement: Supplementary file 3 — Description of Additional Supplementary Files [file 41467_2021_25115_MOESM3_ESM.pdf]

Description of Additional Supplementary Files

File Name: Supplementary Movie 1

Description: intracolonic injection of OVA

File Name: Supplementary Data 1

Description: Prelavence of mouse gut bacteria in different compartments of the gut

File Name: Supplementary Data 2

Description: Differences in gut microbiome distribution in small and large intestine (LefSe and LDA analysis)

File Name: Supplementary Data 3

Description: CytOF panel imunophenotyping on CD45+

File Name: Supplementary Data 4

Description: CytOF panel imunophenotyping of Dendritic cells

File Name: Supplementary Data 5

Description: RNA-seq comparisson on Colon OT-II T cell vs non-OT-II

File Name: Supplementary Data 6

Description: Donor Information
