# Supplementary material for: PD-L1+ and XCR1+ dendritic cells are region-specific regulators of gut homeostasis
Source: Nat Commun. 2021 Aug 13;12:4907. doi: 10.1038/s41467-021-25115-3 (PMC8363668; doi:10.1038/s41467-021-25115-3)
Supplement: Supplementary file 9 — Supplementary Data 6 [file 41467_2021_25115_MOESM9_ESM.pdf]

Supplementary Table 6: Donor Information

Patient ID	Age	Gender	CHC
P1	58	Male	not present
P2	50	Female	Hypertension
P3	55	Male	not present
P4	59	Male	not present
P6	57	Female	not present
P7	66	Male	not present
P8	62	Male	Hypertension
P9	54	Male	Hypertension
P10	55	Male	not present
P11	53	Female	not present

CHC= Chronic health condition
