# Supplementary material for: PD-L1+ and XCR1+ dendritic cells are region-specific regulators of gut homeostasis
Source: Nat Commun. 2021 Aug 13;12:4907. doi: 10.1038/s41467-021-25115-3 (PMC8363668; doi:10.1038/s41467-021-25115-3)
Supplement: Supplementary file 11 — Reporting Summary [file 41467_2021_25115_MOESM11_ESM.pdf]

nature research

Corresponding author(s): Thais Garcias Moreira

Last updated by author(s): 07/01/2021

Reporting Summary

Nature Research wishes to improve the reproducibility of the work that we publish. This form provides structure for consistency and transparency in reporting. For further information on Nature Research policies, see our Editorial Policies and the Editorial Policy Checklist.

Statistics

For all statistical analyses, confirm that the following items are present in the figure legend, table legend, main text, or Methods section.

n/a	Confirmed	
☐	☒	The exact sample size (n) for each experimental group/condition, given as a discrete number and unit of measurement
☐	☒	A statement on whether measurements were taken from distinct samples or whether the same sample was measured repeatedly
☐	☒	The statistical test(s) used AND whether they are one- or two-sided<br>*Only common tests should be described solely by name; describe more complex techniques in the Methods section.*
☐	☒	A description of all covariates tested
☐	☒	A description of any assumptions or corrections, such as tests of normality and adjustment for multiple comparisons
☐	☒	A full description of the statistical parameters including central tendency (e.g. means) or other basic estimates (e.g. regression coefficient) AND variation (e.g. standard deviation) or associated estimates of uncertainty (e.g. confidence intervals)
☐	☒	For null hypothesis testing, the test statistic (e.g. F, t, r) with confidence intervals, effect sizes, degrees of freedom and P value noted<br>*Give P values as exact values whenever suitable.*
☒	☐	For Bayesian analysis, information on the choice of priors and Markov chain Monte Carlo settings
☐	☒	For hierarchical and complex designs, identification of the appropriate level for tests and full reporting of outcomes
☒	☐	Estimates of effect sizes (e.g. Cohen's d, Pearson's r), indicating how they were calculated

Our web collection on statistics for biologists contains articles on many of the points above.

Software and code

Policy information about availability of computer code

Data collection	BD Facs DIVA was used to collect flow cytometry data. CyTOF data was collect on Helios platform.
Data analysis	For flow cytometry analysis , Flowjo version 10.5.3 was used. For mass cytometry analysis we used Cytobank Software version 6.2.<br>For microbiome data analysis , we used open source software from QIIME2 as well as the open source software LEfSe (LEfSe, Version 1) on the Galaxy Browser and have described the parameters used in the methods section<br>A ImageJ macro code was used to batch process images from Immunofluorescence and Immunohistochemistry<br>For RNA-seq analysis data were demultiplexed and provided by the Broad Institute in FASTQ format. Reads were quantified at the transcript level using Salmon against an Ensembl catalog, and aggregated to the gene level using tximport<br>For comparison against the (GSE121811) and (GSE130201) gene-level data, raw gene counts resulting from each experiment were compiled, normalized using the median of ratios method via DESeq2, and filtered for low abundance.<br>Gene groups for gene set enrichment analysis were selected from MsigDB (https://www.gsea-msigdb.org/gsea/msigdb)

For manuscripts utilizing custom algorithms or software that are central to the research but not yet described in published literature, software must be made available to editors and reviewers. We strongly encourage code deposition in a community repository (e.g. GitHub). See the Nature Research guidelines for submitting code & software for further information.

Data

Policy information about availability of data

All manuscripts must include a data availability statement. This statement should provide the following information, where applicable:

- Accession codes, unique identifiers, or web links for publicly available datasets
- A list of figures that have associated raw data
- A description of any restrictions on data availability

Microbiome and RNA-seq data have been uploaded to a publicly available data base from the National Center for Biotechnology Information (NCBI) in the short read archive (SRA) under the BioProject number PRJNA733716
https://www.ncbi.nlm.nih.gov/bioproject/PRJNA733716
Batch analysis of intestinal DC using ImageJ code can be found at https://doi.org/10.5281/zenodo.4876494

Field-specific reporting

Please select the one below that is the best fit for your research. If you are not sure, read the appropriate sections before making your selection.

[x] Life sciences [ ] Behavioural & social sciences [ ] Ecological, evolutionary & environmental sciences

For a reference copy of the document with all sections, see nature.com/documents/nr-reporting-summary-flat.pdf

Life sciences study design

All studies must disclose on these points even when the disclosure is negative.

Sample size	Most studies in our manuscript are collated from 2-3 experiments with N=3-5.<br>For Lamina Propria experiments samples were pooled from 3-5 mice and final N=5 were used.<br>Sample size was determinated based on cell recovery efficiency considering the 3R (Replacement, Refinement and Reduction) in order to alleviate unnecessary animal suffering. Guidelines/ Policies were based on the American Association for Laboratory Animal Science (AALAS) and Brigham and Women's Hospital Institutional Animal Care and Use Committee (IACUC).
Data exclusions	Differential analysis in RNA-seq data was performed using DESeq2 with a false discovery rate cutoff of 5%<br>Microbiota samples surveyed by 16S RNA sequences with less than 1000 reads were removed from analysis.
Replication	Experiments was repeated successfully at least 2 times.
Randomization	Randomization was not undertaken in this study as mouse isogenic strains (identical, or near identical genotypes) were used and healthy subjects (non-diseased) patients were selected (eligibility criteria).
Blinding	Genotypes were known to the experimenters. In disease condition, (histopathology, cytometry/Mass experiments), the investigator was blinded to group allocation. During steady-state condition experiments (flow/mass cytometry, RNA-seq, iRT-PCR, in vitro experiments, IF and IHC staining, microbiome analysis) and for human studies, experimenters were blinded to gut-region.

Reporting for specific materials, systems and methods

We require information from authors about some types of materials, experimental systems and methods used in many studies. Here, indicate whether each material, system or method listed is relevant to your study. If you are not sure if a list item applies to your research, read the appropriate section before selecting a response.

Materials & experimental systems

n/a	Involved in the study	
[ ]	[x]	Antibodies
[x]	[ ]	Eukaryotic cell lines
[x]	[ ]	Palaeontology and archaeology
[ ]	[x]	Animals and other organisms
[ ]	[x]	Human research participants
[x]	[ ]	Clinical data
[x]	[ ]	Dual use research of concern

Methods

n/a	Involved in the study	
[x]	[ ]	ChIP-seq
[ ]	[x]	Flow cytometry
[x]	[ ]	MRI-based neuroimaging

Antibodies

Antibodies used	Flow cytometry (mouse)

Foxp3 MF-14 1:100 Alexa Fluor 488 Biolegend
CD25 3C7 1:100 FITC Biolegend
CD209b LWC06 1:150 FITC Biolegend
anti-GFP FM264G 1:100 Alexa Fluor 488 Biolegend
Ly-6C Hk1.4; 1:100 Alexa Fluor 488 Biolegend
CD11c N418 1:250 PE Biolegend
CX3CR1 SA011F11 1:250 PE Biolegend
Nk1.1 PK136 1:300 PerCP-Cy5.5 Biolegend
B220 RA3-6B2 1:300 PerCP-Cy5.5 Biolegend
Ly-6G 1A8 1:300 PerCP-Cy5.5 Biolegend
CD3e 145-2C11 1:300 PerCP-Cy5.5 Biolegend
CD19 6D5 1:300 PerCP-Cy5.5 Biolegend
CD45 30-F11 Q31-378 APC Biolegend
TLR3 1F8 1:250 APC Biolegend
CD86 GL-1; 1:400 APC Biolegend
IFN-g XMG1.2 1:200 APC Thermo Fischer
RORgt B2D 1:200 APC Thermo Fischer
CD103 2E7 1:400 APC Thermo Fischer
IFN-g TC11-18H10 1:250 BUV395 BD Bioscience
RORgt Q31-378 1:200 BV421 BD Bioscience
Ly-6G 1A8; 1:350,BV421 BD Bioscience
Il-17A TC11-18H10; 1:200, BV421 BD Bioscience
CD45 30-F11 1:350 Alexa fluor 700 Biolegend
CD19 6D5 1:300 APC-Cy7 Biolegend
B220 RA3-6B2 1:300 APC-Cy7 Biolegend
CD90.1 53-2.1 1:300 APC-Cy7 Biolegend
CD62L MEL-14 1:300 APC-Cy7 Biolegend
CD44 IM7 1:300 PE-Cy7 Biolegend
CD101 Moushi101 1:150 PE-Cy7 Thermo Fischer
CD172/Sirpa P84 1:200 PE-Cy7 Biolegend
IL-17A 7B7 1:150 PE-Cy7 Thermo Fischer
Ly-6G 1A8; 1:500 PE-dazzle Biolegend
T-bet 4B10; 1:200 PE-dazzle Biolegend
Siglec-f E50-2440 1:400 BV421 BD Bioscience
CD274/PD-L1 10F.9G2 1:200 BV421 Biolegend
CD8 53-6.7 1:300 BV711 BD Bioscience
CD103 M290 1:300 BV711 BD Bioscience
CD103 M290 1:250 BV786 BD Bioscience
CD4 GK1.5 1:300 Alexa Fluor700 Biolegend
CD4 RM4-5 1:350 BV605,BV786,PerCP-Cy5.5 BD Bioscience
rat-anti mouse CD16/32; 2.4G2 1:100 NA BD Bioscience
F4/80 BM8 1:300 BV605 Biolegend
CD64 X54-5/7.1 1:300 BV605 Biolegend
CD62L MEL-14 1:250 APC-Cy7 Biolegend
CD44 IM 1:250 PE-Cy7 Biolegend
CD25 3C7 1:100 FITC Biolegend
CD45.1 A20 1:300 APC Biolegend
Vα2 B20.1 1:400 APC Biolegend
Vβ5 MR9-4 1:300 PE Biolegend
Flow cytometry (human)
HLA-DR L243 1:300 APC-Cy7 Biolegend
CD11c B-ly6 1:200 PE BD Bioscience
CD3 UCHT1 1:300 PerCP-C5.5 BD Bioscience
CD19 HIB19 1:300 PerCP-C5.5 BD Bioscience
CD14 M5E2 1:300 PerCP-C5.5 BD Bioscience
CD1c L161 1:300 PE-Cy7 Biolegend
CD274/PD-L1 MIH2 1:100 FITC BD Bioscience
XCR1 S15046E 1:200 BV421 Biolegend
CD141 1A4 1:300 APC BD Bioscience
Imunofluorescence and Imunohistochemistry*
E-cadherin 4A2 1:100 unconjugated CST
CD141 E7Y9P 1:1000 unconjugated CST
CD1c EPR23189-196 1:250 unconjugated Abcam
CD11c D3V1E 1:100 unconjugated CST
XCR1 D2F8T 1:100 unconjugated CST
PD-L1 E1L3N® 1:500 unconjugated CST
CD11c* D1V9Y 1:100 unconjugated CST
CytOF panel imunophenotyping on CD45+

CD45 30-F11 1:100 141pr LMA Harvard CyTOF Consortium
CD115 AFS98 1:100 142Nd LMA Harvard CyTOF Consortium
TER-119 TER-119 1:100 143Nd LMA Harvard CyTOF Consortium
CD4 RM4-5 1:100 145Nd LMA Harvard CyTOF Consortium
CD11c N418 1:100 146Nd Fluigidm
Ly6G 1A8 1:100 148Nd LMA Harvard CyTOF Consortium
CD274 10F.9G2 1:100 149Sm LMA Harvard CyTOF Consortium
B220 RA3-6B2 1:100 151Eu LMA Harvard CyTOF Consortium
CD3 145-2C11 1:100 152Sm LMA Harvard CyTOF Consortium
CD172a P84 1:100 153Eu LMA Harvard CyTOF Consortium
CD103 A2F10 1:100 154Sm LMA Harvard CyTOF Consortium
NK1.1 PK136 1:100 155Gd LMA Harvard CyTOF Consortium
CD24 M1/69 1:200 156Gd LMA Harvard CyTOF Consortium
CD205 NLDC-145 1:100 158Gd LMA Harvard CyTOF Consortium
CD39 Duha59 1:100 159Tb LMA Harvard CyTOF Consortium
CD11b M1/70 1:100 160gd Fluigidm
CD26 H194-112 1:100 161Dy Biolegend Purified anti-mouse CD26 (DPP-4)
CD135 A2F10 1:100 162Dy LMA Harvard CyTOF Consortium
XCR1 ZET 1:100 163Dy Biolegend Purified anti-mouse/rat XCR1 Antibody
CD8a 53-6.7 1:100 164Dy LMA Harvard CyTOF Consortium
CCR2 SA203G11 1:100 165Ho LMA Harvard CyTOF Consortium
LAP TW7 1:100 166Er customized CyTOF Consortium
CD64 x54-5/7.1 1:100 168Er LMA Harvard CyTOF Consortium
CX3CR1 SA011F11 1:100 169Tm LMA Harvard CyTOF Consortium
CD40 MR1 1:100 170Er LMA Harvard CyTOF Consortium
CD209 MMD3 1:100 171Yb Biolegend Purified anti-mouse CD209a (DC-SIGN)
TCRgd GL3 1:100 172Yb LMA Harvard CyTOF Consortium
CD69 H1.2F3 1:100 173Yb Biolegend Purified anti-mouse CD370 (CLEC9A, DNGR1)
I-A/I-E (MHC II) M5/114.15.2 1:200 174Yb LMA Harvard CyTOF Consortium
F4/80 BM8 1:100 175Lu LMA Harvard CyTOF Consortium
CCR7 4B12 1:100 176Yb LMA Harvard CyTOF Consortium
CytOF panel imunophenotyping of Dendritic cells
CD45 30-F11 1:100 89Y Fluigidm
CD44 IM7 1:100 113In LMA Harvard CyTOF Consortium
CD4 RM4-5 1:100 115In LMA Harvard CyTOF Consortium
S100A4 S100A4 1:100 141Pr Biolegend Purified anti-S100A4
CD11b AFS98 1:100 142Nd LMA Harvard CyTOF Consortium
TER-119 TER-119 1:100 143Nd LMA Harvard CyTOF Consortium
CD115 AFS98 1:100 144Nd LMA Harvard CyTOF Consortium
CD45RB RA3-6B2 1:100 145Nd Fluigidm
CD11c N418 1:100 146Nd LMA Harvard CyTOF Consortium
CD36 HM36 1:100 147Sm Fluidigm cat#
CD317 927 1:100 148Nd LMA Harvard CyTOF Consortium
CD274 10F.9G2 1:100 149Sm LMA Harvard CyTOF Consortium
CD24 M1/69 1:200 150Nd Fluigidm
CD64 X54-5/7.1 1:100 151Eu Fluigidm
CD3 145-2C11 1:100 152Sm LMA Harvard CyTOF Consortium
CD172a P82 1:100 153Eu LMA Harvard CyTOF Consortium
TCRgd 2.00E+07 1:100 154Sm LMA Harvard CyTOF Consortium
NK1.1 PK136 1:100 155Gd LMA Harvard CyTOF Consortium
CD272/BTLA 6F7 1:100 156Gd Fluigidm
CD205 NLDC-145 1:100 158Gd LMA Harvard CyTOF Consortium
CD206 C068C2 1:100 159Tb LMA Harvard CyTOF Consortium
CD207 M1/70 1:100 160Gd LMA Harvard CyTOF Consortium
CD26 H194-112 1:100 161Dy Biolegend Purified anti-mouse CD26 (DPP-4)
CD103 A2F10 1:100 162Dy LMA Harvard CyTOF Consortium
XCR1 ZET 1:100 163Dy Biolegend Purified anti-mouse/rat XCR1 Antibody
CD8a 53-6.7 1:100 164Dy LMA Harvard CyTOF Consortium
TLR3 poly 1:100 165Ho LMA Harvard CyTOF Consortium
LAP TW7 1:100 166Er LMA Harvard CyTOF Consortium
CD25 3C7 1:100 167Er LMA Harvard CyTOF Consortium
c-Kit x54-5/7.1 1:100 168Er Fluigidm
Cx3Cr1 SA011F11 1:100 169Tm LMA Harvard CyTOF Consortium
Sigle-c 3/23 1:100 170Er LMA Harvard CyTOF Consortium
CD209 MMD3 1:100 171Yb Biolegend Purified anti-mouse CD209a (DC-SIGN)
Epcam G8.8 1:100 172Yb LMA Harvard CyTOF Consortium
Cleac9a 7H11 1:100 173Yb Biolegend Purified anti-mouse CD370 (CLEC9A, DNGR1)
I-A/I-E (MHC II) M5/114.15.2 1:200 174Yb LMA Harvard CyTOF Consortium

	F4/80 BM8 1:100 175Lu LMA Harvard CyTOF Consortium<br>CCR7 1:100 176Yb LMA Harvard CyTOF Consortium
Validation	All antibodies employed were commercial available in which validations were previously performed by manufacture. LMA Harvard CyTOF Consortium performed conjugation validation for customized metal-labeling antibodies.<br>Additionally, Fluoresce Minus One (FMO) and Metal Minus One (MMO) were used for staining validation in the experiments when necessary. The use of samples from Knockout mice and reporter mice were used to validate antibodies when necessary.<br>The dilutions provided represents the optimal concentration as determined in this study.

Animals and other organisms

Policy information about studies involving animals; ARRIVE guidelines recommended for reporting animal research

Laboratory animals	XCR1DTA mice were generated by cross-breeding B6.Cg-Xcr1<tm4(cre)Ksho> (RBRC09929) and B6.129P2-Gt(ROSA)26Sortm1(DTA)Lky/J (Jax 009669)<br>XCR1Venus mice were generated by breeding B6.Cg-Xcr1<tm1Ksho> (RBRC09486)<br>PD-L1fl/fl CD11cCre mice were provided by Dr. Arlene Sharpe and PD-L1-/- mice from Dr. Murugaiyan Gopal from BWH/HMS.<br>Itgax reporter mice were generated by breeding Itgax-Cre mice and mT/mG mice (B6.129(Cg)-Gt(ROSA)26Sortm4(ACTB-tdTomato,-EGFP)Luo/J (Jax# 007676).<br>S100a4 reporter mice were generated by breading B6.C-Tg(S100a4-cre)1Egn/JhrsJ (Jax 030644) and mT/mG mice<br>C57BL/6J mice were purchased from Jackson laboratory (JAX 000664).<br>For adoptive transfer experiments, recipient mice CD45.1B6.SJLPtprca Pepcb/BoyJ (002014) and donor mice B6.Cg Tg(TcraTcrb) 425Cbn/J was purchased by Jax(004194).<br>Mice were housed under specific pathogen-free conditions at Hale Building for Transformative Medicine at Brigham and Women's Hospital according to the animal protocol guidelines of the Committee on Animals of BWH. Light Cycle from 7am- lights on, 7pm- lights off , Temperature of 68-75F, humidity of 35-65%. Mice purchased from the Jackson laboratory were acclimated in the local animal facility for at least one week prior to study initiation and genders were matched for each experiment. Otherwise specified, all mice used were 8-10 weeks old at the initiation of study.
Wild animals	Does not involve wild animal
Field-collected samples	Does not involve field-collected samples
Ethics oversight	All animal studies were approved by Committee on Animals of BWH; IACUC 2016N000230

Note that full information on the approval of the study protocol must also be provided in the manuscript.

Human research participants

Policy information about studies involving human research participants

Population characteristics	>18 years old, male and female (Supplementary table 5)
Recruitment	Human duodenal and colonic biopsies from the same patient were obtained at Biogastro in Belo Horizonte, Brazil<br>All donors were free of chronic intestinal disease (Table S5)<br>Subjects were selected using continuous order of appointment in the clinics.<br>Paired-up sample/analysis was used.
Ethics oversight	(CAAE No. 35312820.7.0000.5149, COEP, UFMG)<br>Experiments were performed following good clinical practice guidelines and declaration of Helsinki. Consent form were signed voluntarily by each patient prior to sample collection.

Note that full information on the approval of the study protocol must also be provided in the manuscript.

Flow Cytometry

Plots

Confirm that:

- [x] The axis labels state the marker and fluorochrome used (e.g. CD4-FITC).
- [x] The axis scales are clearly visible. Include numbers along axes only for bottom left plot of group (a 'group' is an analysis of identical markers).
- [x] All plots are contour plots with outliers or pseudocolor plots.
- [x] A numerical value for number of cells or percentage (with statistics) is provided.

Methodology

Sample preparation	Surface staining was performed according to standard procedures at a density of 0.3-1 x 106 cells per 50 µl, and volumes were scaled up accordingly. Fc block (rat-anti mouse CD16/32; clone 2.4G2; BD Biosciences) was used at 1:100 dilution. Cells isolated from human biopsies were incubated with Human Fc Block (1:100; BD Bioscience). For Foxp3 intracellular staining,

	Foxp3 staining kit (Thermo Fisher) was used as per manufacturer's instructions. For cytokine staining, mouse intracellular cytokine staining kit (BD Biosciences) was used.
Instrument	Flow cytometry sample acquisition was performed in BD Fortessa, BD Symphony and FACScanto (BD Biosciences).
Software	FLowJo (Tree STar Inc)
Cell population abundance	sorted cells were counted. For DC in vitro cocuture, 1000 cells were plated per well. For RNA-seq 1000cells were collected directly into eppendorf tubes containing lysis buffer.
Gating strategy	Gating strategy for Mass cytometry (provided as supplementary information). Metal minus one (MMO) was applied for gating.<br>Gating strategy for Flow cytometry involved (FSC vs SSC) size gating, followed by removal of duplets. Further viability and CD45 gating was applied. Fluorescence minus one (FMO) was applied for gating

☒ Tick this box to confirm that a figure exemplifying the gating strategy is provided in the Supplementary Information.
